# Structure and evolution of *Apetala3*, a sex-linked gene in *Silene latifolia*

**DOI:** 10.1186/1471-2229-10-180

**Published:** 2010-08-18

**Authors:** Radim Cegan, Gabriel AB Marais, Hana Kubekova, Nicolas Blavet, Alex Widmer, Boris Vyskot, Jaroslav Doležel, Jan Šafář, Roman Hobza

**Affiliations:** 1 Laboratory of Plant Developmental Genetics, Institute of Biophysics, Academy of Sciences of the Czech Republic, v.v.i.Kralovopolska 135, CZ-612 65 Brno, Czech Republic; 2 Department of Plant Biology, Faculty of Agronomy, Mendel University in Brno, Zemedelska 1, CZ-613 00 Brno, Czech Republic; 3 Laboratoire de Biométrie et Biologie Evolutive (UMR 5558); CNRS University Lyon 1, Bat. Gregor Mendel, 16 rue Raphaël Dubois, 69622, Villeurbanne Cedex, France; 4 Institute of Integrative Biology, Plant Ecological Genetics, ETH Zurich, Universitaetstrasse 16, 8092 Zurich, Switzerland; 5 Laboratory of Molecular Cytogenetics and Cytometry, Institute of Experimental Botany, Academy of Sciences of the Czech Republic, v.v.i. Sokolovska 6, 772-00, Olomouc, Czech Republic

## Abstract

**Background:**

The evolution of sex chromosomes is often accompanied by gene or chromosome rearrangements. Recently, the gene *AP3 *was characterized in the dioecious plant species *Silene latifolia*. It was suggested that this gene had been transferred from an autosome to the Y chromosome.

**Results:**

In the present study we provide evidence for the existence of an X linked copy of the *AP3 *gene. We further show that the Y copy is probably located in a chromosomal region where recombination restriction occurred during the first steps of sex chromosome evolution. A comparison of X and Y copies did not reveal any clear signs of degenerative processes in exon regions. Instead, both X and Y copies show evidence for relaxed selection compared to the autosomal orthologues in *S. vulgaris *and *S. conica*. We further found that promoter sequences differ significantly. Comparison of the genic region of *AP3 *between the X and Y alleles and the corresponding autosomal copies in the gynodioecious species *S. vulgaris *revealed a massive accumulation of retrotransposons within one intron of the Y copy of *AP3*. Analysis of the genomic distribution of these repetitive elements does not indicate that these elements played an important role in the size increase characteristic of the Y chromosome. However, *in silico *expression analysis shows biased expression of individual domains of the identified retroelements in male plants.

**Conclusions:**

We characterized the structure and evolution of *AP3*, a sex linked gene with copies on the X and Y chromosomes in the dioecious plant *S. latifolia*. These copies showed complementary expression patterns and relaxed evolution at protein level compared to autosomal orthologues, which suggests subfunctionalization. One intron of the Y-linked allele was invaded by retrotransposons that display sex-specific expression patterns that are similar to the expression pattern of the corresponding allele, which suggests that these transposable elements may have influenced evolution of expression patterns of the Y copy. These data could help researchers decipher the role of transposable elements in degenerative processes during sex chromosome evolution.

## Background

Sex chromosomes evolved independently many times in both animals and plants [1]. The initial steps of their evolution, including the genetic degeneration of the non-recombining Y or W chromosomes (which are analogous to Y chromosomes), have received great interest from geneticists. To date, most of our knowledge about sex chromosome evolution stems from a few animal systems with evolutionary old sex chromosomes [2]. However, evolutionarily young sex chromosomes are needed to investigate the early steps in sex chromosome evolution. Such sex chromosomes can be found in plants [3,4]. Although the majority of plants are cosexuals, forming either bisexual flowers (hermaphrodites) or unisexual flowers of both sexes on one individual (monoecy), dioecious plant species (with separate sexes) have evolved multiple times in different plant lineages [5]. The majority of dioecious plant species lack morphologically distinguishable sex chromosomes. However, well differentiated heteromorphic sex chromosomes were described in *Rumex acetosa*, *Cannabis sativa *and *Silene latifolia*. The latter has become a model species for investigations into the evolution of sex chromosomes in plants.

*Silene latifolia *Poiret (syn. *Melandrium album *Garcke, syn. *Melandrium pratense *Roehl.) is a strictly dioecious, perennial herb of the *Caryophyllaceae *family. The sex of individual plants is genetically determined by sex chromosomes that were first described independently by Blackburn [6] and Winge [7]. Females are homogametic with a pair of X chromosomes, while the males are heterogametic, XY [8]. The X and Y chromosomes are about 1.4-fold and 2-fold larger than the largest autosome, respectively [9]. Therefore, they contribute substantially to the large genome size of the species and to the slightly larger genome size in males than in females [10]. The Y chromosome in *S. latifolia *seems to lack some essential genes present on the X, since plants are not viable unless they have at least one X chromosome [11]. By analyzing hermaphroditic mutants and their progeny, Westergaard [12] showed that all independently derived hermaphrodites had deletions in one arm of the Y chromosome. From the studies on deletion mutants, Westergaard [13] concluded that one arm of the Y chromosome contains gene(s) for anther maturation, while the other arm has gene(s) suppressing carpel development, and additional genes located close to the centromere stimulate early stages of stamen development [13]. More recently, molecular markers in combination with a panel of deletion mutants were used to create a detailed map of the Y chromosome [14-16].

Gene and genome duplications have been recognized as major forces driving the evolution of animal and plant genomes. Two basic processes can cause duplication of genes. The first process, segmental duplication, keeps the structure of a gene (exon-intron order, cis regulatory sequences) in its original constitution. The duplicated copy of the gene maintains expression patterns similar to the original copy. The second process, retrotransposition, often generates non-functional gene copies that lack regulatory elements and introns [17,18].

The evolution of sex chromosomes is a complex genetic and epigenetic process [1], which is often accompanied by structural rearrangements and accumulation of repetitive DNA in non-recombining regions. Moreover, intensive gene turnover within sex chromosomes is reflected by a high number of retroposed genes both on X and Y chromosomes [19,20]. It is known that over the course of *S. latifolia *sex chromosome evolution, many repetitive elements have accumulated on the Y chromosome [21]. However, we still lack information about which elements are linked to degenerative processes in Y chromosome evolution by either genetic or epigenetic mechanisms [22], and little is known about the structural and functional role of repetitive DNA in Y linked genic regions of this plant.

Here we unravel the structure and evolution of a sex linked gene, *SlAP3*, first reported as having originated by duplication from autosomes to the Y chromosome in *S. latifolia *[23]. Since *SlAP3Y *is located close to the oldest stratum (4.5-7 MY) in the Y chromosome, this gene is a candidate to be affected by various degenerative processes [24,25]. In our study, we did not find evidence for a duplication event in the case of this gene. Instead, we identified a new pair of sex linked alleles with no evidence for autosomal paralogues. We demonstrated the accumulation of retrotransposon sequences in an intron region of the Y linked allele. We further analyzed expression patterns of individual elements identified in the Y copy of *SlAP3 *gene to reveal their role in Y chromosome evolution.

## Results

### Identification of genomic clones for *APETALA3 *(*AP3*) gene

*S. latifolia *and *S. vulgaris *BAC libraries were screened with *SlAP3A *and *SlAP3Y *gene derived probes prepared using the sequences and primers from Matsunaga *et al. *[23]. Positively hybridizing clones were selected, and the presence of the target gene was verified by PCR including sequencing of PCR products. In total, we identified four clones containing the *AP3 *gene in the *S. latifolia *BAC library. Two clones contained the presumed autosomal *SlAP3A *copy (246/K15, 251/L13) and the other two contained *SlAP3Y *(30/L22 and 253/J6). Four copies of an orthologue, which we called *SvAP3*, were identified in the *S. vulgaris *BAC library. Both BACs containing the *SlAP3Y *copy were selected for further complete BAC sequencing. For the *SlAP3A *and *SvAP3 *BAC clones, we isolated DNA, digested it with *HindIII *and conducted a Southern blot hybridization. The original probes from the BAC library screening were used for the hybridization. Subsequent experiments revealed no difference in hybridization patterns within BAC groups (*SlAP3A, SvAP3*) (Additional file 1, Figure S1). Differences in signal intensity of individual hybridizing BACs are due mainly to the different sizes of BAC inserts, which prevented us from using equimolar amounts during gel loading. Based on complete sequence similarity (after sequencing of PCR products with the same primers as used for probe preparation) and an identical hybridization profile, we randomly selected BAC clones 91/M20 (containing *SvAP3*), 251/L13 (*SlAP3A*) and 30/L22, 253/J6 (*SlAP3Y*) for a more detailed analysis. The data obtained strongly suggest that there is just one allelic pair of the *SlAP3 *gene in both the *S. latifolia *and *S. vulgaris *genomes.

### Linkage mapping by PCR on microdissected chromosomes

**Figure 1:**
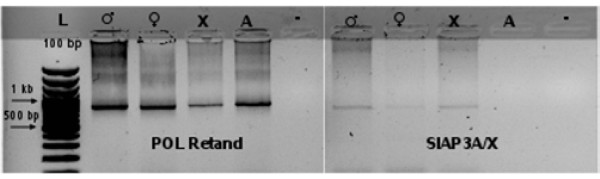
**PCR on microdissected chromosomes**. POL Retand primers were used as a positive control (present in all chromosomes) and primers for *SlAP3A/X *K-domain were used for sex chromosomes localization. The template DNA is indicated in the figure (size marker (L) 100 bp, male genomic DNA (♂) female genomic DNA (♀) microdissected X chromosomes (X), microdissected autosomes (A) and negative control (no template). PCR products were subjected to electrophoresis on 1% agarose gel and stained with ethidium bromide.

To confirm the linkage of individual *SlAP3 *alleles to specific chromosomes, we microdissected and separated X chromosomes and autosomes in *S. latifolia *(Additional file 2, Figure S2) and ran PCR using these chromosomes as templates. We used primers for POL sequence of the *Retand *retroelement [26] as a positive control. Surprisingly, PCR with primers for the *SlAP3A *K-domain resulted in a product only with genomic DNA of both sexes and using microdissected X chromosomes as a template (figure 1). We did not obtain any PCR product from microdissected autosomes, an observation which did not confirm the presumed presence of a copy of the *SlAP3A *gene on an autosome. These observations demonstrate that *SlAP3A*, originally described as an autosomal copy in Matsunaga *et al. *[23], is in fact an X linked allele of the *SlAP3Y *gene, which we call from here on *SlAP3X*.

### Genomic organization

**Figure 2:**
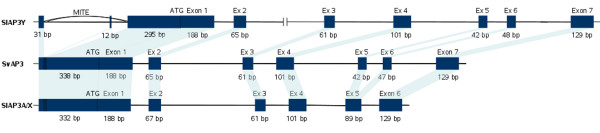
**Alignment of a promoter and coding region of *SlAP3X, SlAP3Y and SvAP3 *genes**. Rectangles represent exon regions of the genes. Corresponding coding sequences are indicated

BAC clones 251/L13, 30/L22, 253/J6 and 91/M20 were purified and sequenced. We identified a copy of the *AP3 *gene in all the BACs sequenced [GenBank: HQ113124, HQ113125, HQ113126]. Comparison with mRNA for *S. latifolia SlAP3A *[GenBank: AB090863] and *SlAP3Y *[GenBank: AB090864] revealed seven exons for *SlAP3Y *and *SvAP3*, while *SlAP3X *contains only six exons (figure 2).

**Figure 3:**
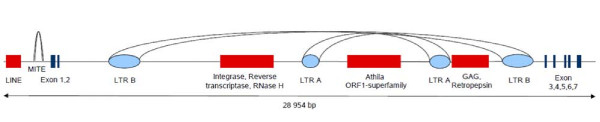
**The structure of *SlAP3Y *gene**. Red rectangles represent coding domains of retrotransposons. Blue rectangles are individual exons of *SlAP3Y*. Ovals represent long terminal repeats.

The size of individual introns differed only slightly among the different copies of *AP3 *(figure 2). The only exception was a very large intron 2 (23,855 bp) in the *SlAP3Y *allele. We found that this intron contains two different retroelements that are shuffled into each other. The first retroelement contains a pair of LTRs, reverse transcriptase, RNaseH, integrase and gag gene, while the sequence of the second retroelement is incomplete and composed of LTRs and ORF1 product from Athila ORF-1 family (figure 3).

### Genomic distribution of repetitive DNA in *SlAP3Y *intron

All coding domains (LINE, reverse transcriptase, integrase) and LTRs were used as probes for FISH on mitotic metaphase chromosomes (Additional file 3, Figure S3). Analysis of the distribution of individual signals did not indicate a specific role of these particular retroelements in Y chromosome evolution (figure S3). FISH analysis was also conducted with the tandemly arrayed repetitive Y promoter motive. There was no signal both after standard and low stringency FISH experiments, indicating a low abundance of this repetitive satellite motif in the *S. latifolia *genome.

### Expression analysis of Y linked retroelements

**Figure 4:**
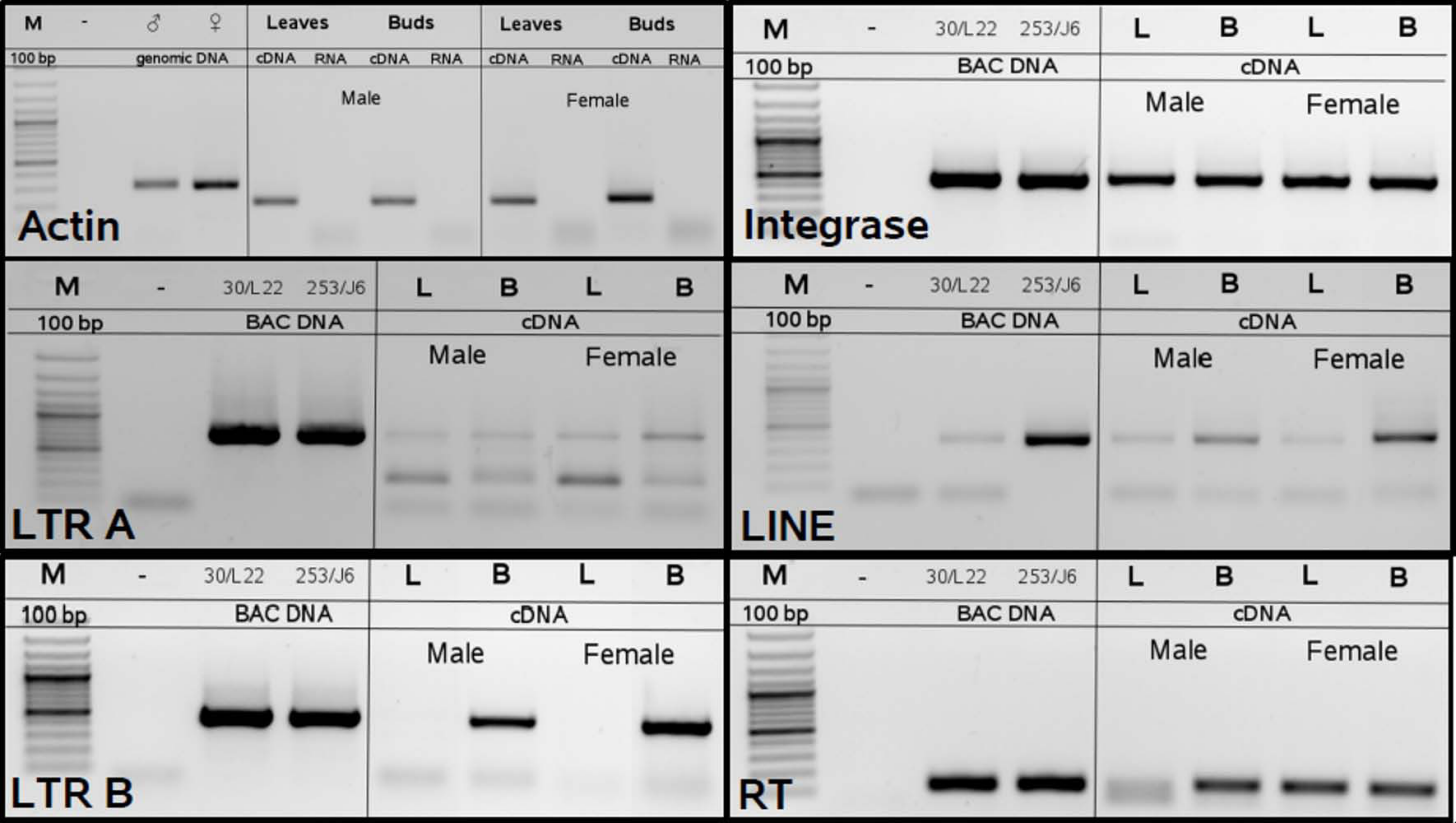
**RT-PCR analysis**. Actin, LTR A, LTR B, integrase, reverse transcriptase (RT) and LINE primers were used. Genomic DNA with actin primers and BAC DNA with element specific primers were used as a positive control. Actin reveals a different sized PCR product when genomic DNA is used as a template than when cDNA is used as a template due to intron excision, and so can be used as an internal control for purity of RNA used for reverse transcription. Template DNA is indicated in the figure (size marker (M) 100 bp ladder), male genomic DNA (♂), female genomic DNA (♀), BAC DNA (30/L22 and 253/J6) *S. latifolia *male and female cDNA and RNA from leaves (L) and buds (B) and negative control (-, no template). PCR products were subjected to electrophoresis on 1% agarose gel and stained with ethidium bromide.

To reveal activity (expression) of individual repetitive elements linked to *SlAP3Y *and their domains, we conducted RT-PCR experiments (figure 4). Retroelement A (its LTR part) and LINE were expressed in both males and females in different tissues (leaves and buds). Surprisingly, retroelement B had a different pattern of expression between the LTR part and the rest of its genes. Although integrase and RT domain showed similarly to retroelement A, and LINE widespread expression in both sexes and all tissues, the LTR B region was expressed only in the floral buds of both sexes.

We quantified the expression of individual domains of the retroelements localized in the *SlAP3Y *copy by identifying the number of hits with the male and female specific EST database of *S. latifolia*. This database is composed of ca 100,000 reads generated by 454 sequencing (Roche FLX 454 pyrosequencing) using cDNA from male and female buds as a sequencing template. The accuracy of the selected approach was tested by comparing the occurrence of part of the actin gene in both male and female EST databases (Table 1). Internalpart of retroelement B (integrase, reverse transcriptase) revealed a similar number of hits (expression level) in both males and females. LTR A, LTR B and LINE element expression was stronger in males based on the EST database data. LTR B, which has 28× higher expression in males than in females, was the most different.

**Table 1:** Estimation of intensity of expression of different parts of retroelements in intron 2 based on the number of reads in *S. latifolia *cDNA libraries (actin was used as an internal control).

	Male	Female
**LTRA**	29	15
**LTRB**	56	2
**RT (Retroelement B**	9	10
**Integrase (Retroelement B)**	2	1
**LINE**	11	2
**Actin**	7	8

### Promoter structure analysis

By comparing promoter sequence structure, we discovered significant differences in *SlAP3Y *in comparison with *SlAP3X *and *SvAP3*. A part of the *SlAP3Y *promoter is formed by a 6 bp long direct repeat. Both ends of the repeat are bordered by inverted tandem structures that resemble the organization of a MITE element (Additional file 4, Figure S4). GenBank database searches revealed no similarity of this part of the promoter to any known sequence, except for the *MROS1 *gene [27] promoter region [GenBank: AB013446.1]. Although *MROS1 *is not a sex-linked gene, like *SlAP3Y *it is expressed only in males.

### Sequence divergence analysis

In *S. latifolia*, recombination between the sex chromosomes has ceased in three steps, and three groups of genes with different levels of divergence (also called strata) have been identified [25,28]. The level of divergence between *SlAP3 *X and Y copies is about 13% (see Table 2), between the 20% X-Y divergence typical of the stratum 1 genes and the 10% X-Y divergence typical of the stratum 2 genes.

**Table 2:** dN and dS analysis in X/Y, dioecious and non-dioecious species.

dN/dS analysis	dS X-Y	dN X-Y	dN/dS X	dN/dS Y	dN/dS
	*S. latifolia*	*S. latifolia*	*S. latifolia*,	*S. latifolia*,	autosomal
			*S. dioica*,	*S. dioica*,	*S. vulgaris*,
			*S. diclinis*	*S. diclinis*	*S. conica*

**Values**	**0.135 +- 0.035**	**0.055 +- 0.011**	**0.341**	**0.479**	**0.114**

We conducted a dN/dS analysis on the *SlAP3 *sequence to study the possibility of differences in intensity and form of selection in the X and Y copies of this gene (and also with autosomal orthologs). We included all available orthologous sequences from *Silene *species to have as many sequences as possible for the phylogenetic dN/dS analysis, which tends to give more accurate results with more sequences, and to have outgroups (the non-dioecious species *S. vulgaris *and *S. conica*). The results are reported in table 2 and in additional file 5, figure S5. The dN/dS ratios in X and Y sequences among dioecious *Silene *species were not found to be significantly different, which does not provide clear evidence of the Y copy degeneration. However, dN/dS ratios were found to be significantly different (p-value = 0.0149) between dioecious and non-dioecious lineages. The higher ratio in the dioecious lineage suggests that selection has been relaxed in both the X and Y sequences compared to the autosomal copy in *S. vulgaris *and *S. conica *where dN/dS is much lower (see Table 2).

## Discussion

By screening more than five haploid complements of the *S. latifolia *genome, we identified two copies each of *SlAP3Y *and *SlAP3A genes*. The number of identified BAC clones containing variants of the Y and the presumed autosomal copy suggests that *SlAP3 *is a single copy gene. According to Matsunaga *et al. *[23], the Y copy of *SlAP3 *is a paralog of an autosomal allele that was transferred onto the Y chromosome after the beginning of sex chromosome evolution in *Silene*. Although the authors mentioned the existence of introns in both paralogs, genomic sequence data were not presented. To decipher the mechanism of the translocation of *SlAP3 *gene within the *S. latifolia *genome, we isolated and sequenced BAC clones containing *SlAP3 *paralogs. Moreover, we isolated and sequenced *SlAP3 *gene from the *S. vulgaris *genome (a closely related gynodioecious plant without sex chromosomes) and named this gene *SvAP3 *(*Silene vulgaris APETALA3 *gene). Comparison of the orthologs between *S. latifolia *and *S. vulgaris *revealed that the Y copy of *S. latifolia *and the autosomal copy of *S. vulgaris *have seven introns, whereas the X copy of *S. latifolia *contains only six introns. Surprisingly, the promoter region of *SlAP3Y *copy is completely different from both *SvAP3 *and *SlAP3A*.

To study the extent of the translocated autosomal region on the Y chromosome we intended to select low copy markers in the Y BAC clone and to map them on dissected sex chromosomes and autosomes by PCR. However, PCR with the *SlAP3 *specific primers revealed different localization of *SlAP3 *paralogues in the genome than expected. While we were able to amplify *SlAP3 *gene copies using X chromosomes as templates, there was no PCR product when only autosomes served as a template. These observations show that *SlAP3 *is a regular sex-linked gene with X and Y alleles, and that no transfer to the Y chromosome has occurred. Our data suggest different results as shown in Matsunaga *et al. *[23]. The simplest explanation in this case could be use of different techniques for separation of sex chromosomes. Chromosome sorting used by Matsunaga *et al. *[23] is a powerful method which generates large amount of DNA for further experiments. The main disadvantage of this method is that it is subject to impurities even when the chromosomes being sorted are significantly morphologically different, as is the case for *S. latifolia *sex chromosomes and autosomes [29]. Unlike chromosome sorting, laser microdissection is more laborious but produces a pure fraction of selected material [30,31].

Based on comparisons of the X and Y alleles we showed that *SlAP3X *and *SlAP3Y *started to diverge quite early: their divergence at synonymous sites (13%) is close to the maximum X-Y divergence recorded so far for *S. latifolia *sex linked genes, although the mapping of *SlAP3 *on the X chromosome is needed to confirm this. No clear evidence of degeneration of the Y copy has been found in this case. Rather, the dN/dS values for dioecious vs. non-dioecious lineages suggest a subfunctionalization in which X and Y copies have differentially retained their ancestral functions (still coded by one gene in the non-dioecious species), as has been hypothesized for some sex-linked genes in humans [32]. Moreover, a branch-site analysis (Additional file 6, Table S1) revealed no significant evidence for positive selection on the Y sequences (or on the X sequences), which further supports subfunctionalization. Furthermore, the expression patterns of the Y copies and the X copies also suggest subfunctionalization [23]. The observed differences in expression patterns between *SlAP3X *and *SlAP3Y *are due to regulatory differences of particular alleles in males, where both *SlAP3X *and *SlAP3Y *are present. Comparisons of promoter sequences of all three copies of *AP3 *revealed that the autosomal promoter in *S. vulgaris *is identical to the *SlAP3X *promoter. Surprisingly, there is a unique 80 bp promoter region in the *SlAP3Y *copy. The only regulatory sequence that shows similarity with this Y promoter specific structure is a part of the *MROS1 *gene promoter in *S. latifolia *[27]. This gene (localized on autosomes) is also expressed exclusively in males. Although the role of the identified promoter with the same expression pattern of both *SlAP3Y *and *MROS1 *should be verified by e.g. transformation of a plant with "promoter-reporter gene " construct, an efficient transformation protocol is not yet available for *Silene *species. The most prominent evidence suggesting a role of the promoter in sex-specific gene expression regulation is its uniqueness in genome of *S. latifolia*; it appears only in a genic context and mainly co-occurrs with sex specifically expressed genes. Although it is a tandem repeat, it shows no accumulation in *S. latifolia *genome. Even a GenBank search of *Silene *species derived sequences shows no hits with these repetitive DNA fractions.

Our results have implications for intron size evolution in evolving sex chromosomes. We identified a very large intron containing two retrotransposons between the exons 2 and 3 of *SlAP3Y*. A similar phenomenon showing accumulation of different sequences in the Y linked introns was previously reported for some other *S. latifolia *genes [33,34]. Although it is known that different repetitive elements such as microsatellites [35], tandem repeats [36], organellar DNA [37] and different retrotransposons [38,39] have played a prominent role in the formation of sex chromosomes in *S. latifolia*, there is no direct link between the structural role of such elements and their impact on degeneration of the Y chromosome. To assess the role of these repetitive elements in the Y chromosome evolution we ran FISH experiments and expression (RT-PCR and *in silico*) analysis. Although we did not find specific accumulation of the studied elements in the Y chromosome, we observed different expression patterns among different retroelements and in different tissues. RT-PCR data showed that the LTR B domain, unlike other parts of retroelement B, is specifically expressed in the floral buds of both sexes. These data show an increased activation of a specific type of retroelement during early developmental stages. This phenomenon could be linked to meiotic stages of cells in developing buds when the epigenetic status of genetic information is reassembled and the regulatory role of epigenetic mechanisms is suppressed. A higher expression level was found in LTR A, LINE and especially LTR B in male bud tissue based on *in silico *analysis using EST databases. Although it is known that in males, germ line activity of some retroelements is elevated compared to females [40], we suggest several explanations that concern the evolution of sex chromosomes. It is known from recent papers [33,34], that Y linked genes contain more repetitive elements in their introns compared to X alleles. Higher expression of such Y linked retroelements could be a consequence of co-expression of the element with Y linked genes. Biased occurrence of both LTRs identified in the *SlAP3Y *gene in male EST database could also be explained by another aspect of sex chromosomes evolution. Once a retroelement appears in a Y linked gene it follows the same rules for degenerative processes as Y linked genes. Entire retrotransposonal genes could loose their functions by mutation accumulation and the introduction of stop codons. Retrotranscription begins in the LTR region, and thus this part of the retrotransposon is kept in the expressome even after termination of transcription in other parts of the retroelement (gag, pol). It is also known that unpaired DNA caused by retrotransposon insertion into a chromosome is recognized by RNAi machinery and consequently homologous RNA is degraded [41] . Since there is no pairing of chromosomes in non-recombining regions of sex chromosomes, this mechanism is not triggered in this case and expression of retrotransposons is not regulated in males.

Our data further suggest that previously published retroelement types that are accumulated on the Y chromosome [38] are different from those that participate in intron targeting of Y linked genes. Analysis of the Y promoter linked repetitive microsatellite motif within a MITE element reveals a low abundance in *S. latifolia *genome. Paradoxically, the sequences responsible for the large size of sex chromosomes may thus be different from those that have potentially caused degeneration of Y linked genes.

## Conclusions

This study unravels the structure and evolution of *AP3*, a sex linked gene with copies on the X and Y chromosomes in the dioecious plant *S. latifolia*. This gene was previously reported to be located on the autosomes, with one copy having been transferred by duplication to the Y chromosome. Our results provide evidence for the location of copies on X and Y chromosomes, and the absence of this gene on autosomes. Divergence between the X and Y-linked copies both at sequence (promoter and coding regions) and gene expression levels suggests subfunctionalization has been an important process in the evolutionary dynamics of this gene. One intron of the Y-linked allele was invaded by retrotransposons that display sex-specific expression patterns, similar to the expression pattern of the corresponding allele, which suggests transposable elements may have contributed to the evolution of gene expression of this gene

## Methods

### Plant material and isolation of metaphase chromosomes

*Silene latifolia *Garcke and *Silene vulgaris *plant material was obtained from a seed collection of the Institute of Biophysics, Brno. Sterilized seeds were cultured for 2 days in distilled water and then synchronized with aphidicoline (30 mmol/l for 12 h) and oryzalin (15 μmol/l for 4 h). Root tips from germinating seedlings were cut off and enzymatically protoplasted. The protoplasts were briefly fixed in the mixture of ethanol:acetic acid (3:1) to avoid further DNA damage. The mitotic protoplast suspension was dropped on a membrane (for laser microdissection, stained with Giemsa) or on microscope slides (for FISH experiments), where naked chromosomes were released [31].

### BAC library construction and screening

The BAC libraries were constructed from *S. latifolia *male and *S. vulgaris *high molecular weight genomic DNA. Briefly, DNA was digested with *Hind*III enzyme and inserted into a pECBAC1 and pIndigoBAC-5 (Epicentre) vectors, respectively. Clones were then grid in duplicate on Hybond N^+ ^(Amersham, Biosciences) nitrocellulose membrane filters in a 4 X 4 pattern that allowed us to identify well positions and plate numbers of each clone. The filters were incubated and processed as described in Bouzidi *et al. *[42]. The *S. latifolia *BAC library (total of 119,808 colonies) was arrayed on six nylon filters with 18,432 colonies each, and one nylon filter containing 9,216 clones. The average insert-size of the library is 128 kb. The *S*. *vulgaris *BAC library (total of 55,296 clones) was arrayed on three nylon filters with 18,432 colonies each. The average insert-size of the library was 110 kb. Based on nuclear size data by Vagera *et al *[10] and Široký *et al. *[43], we have estimated that coverage of the *S. latifolia *BAC library is 5.327 complements of the male haploid genome and the *S. vulgaris *BAC library is 6.8 complements of the haploid genome.

Screnning was performed by radioactive hybridization with α^32^P and with Prime-It II Random Primer Labelling Kit (Stratagene) according to the manufacturer's protocol. Probes were prepared by PCR amplification of K-box region of *SlAP3A *and *SlAP3Y *(see PCR).BAC DNA was isolated by Large Construct Kit (Qiagen). DNA for PCR reactions was isolated using NaOH/SDS precipitation according to Sambrook and Russel [44].

### DNA amplification

For K-domain of *SlAP3A *and *SlAP3Y *and *SvAP3 *amplification we used primers according to Matsunaga *et al*., [23] and for MEF2A domain primers SVMEF2_F (5'-GGAAGAGGAAAGTTAGAGAT-3'), SVMEF2_R (5'-TGCAATTTGTGGGTGCTAGA-3') with male and female genomic DNA and with DNA of positively hybridized BAC clones. For amplification of conservative domains of retrolelements in the intron 2 of *SlAP3Y *we used the following primers: GAG_IN2_F (5'-CACCCTTGTCGGTTTCAATC-3'), GAG_IN2_R (5'-AGCGGATGCTAAGGAGATCA-3'), RT_IN2_F (5'-CCATCAACTACCCCCATTTG-3'), RT_IN2_R (5'-GTGGATACGGCTAAGGTGGA-3'), Integ_IN2_F (5'-CCTCTTCACCTTGCAACTCC-3'), Integ_IN2_R (5'-TTTGCAACAAGGTGATCTCG-3'), LTRB_IN2_F (5'-CCGAAGTTGGAGACTTTGGA-3'), LTRB_IN2_R (5'-GTTAATCCTCCCGTCCCAAT-3'). Primers for the POL part of the RETAND element were designed according to Kejnovsky *et al. *[26]. PCR conditions were the same for all primer pairs used. The reaction profile included 35 cycles of 45 s at 94°C, 1 min at 61°C and 2 min at 72°C preceded by initial denaturation (4 min at 94°C) and followed by final extension step (10 min at 72°C). PTC-200 (MJ Research) and T3000 (Biometra, Goettingen, Germany) thermal cyclers were used.

### RT-PCR

Total RNA was extracted from young flower buds (0.1-0.4 cm), and leaf tissues using RNA blue (Top-Bio). One microgram of total RNA was treated with RNase-free DNase (Ambion) and then used for cDNA synthesis using High Capacity RNA-to-cDNA Kit (Applied Biosystems) according to manufacturer's protocol. The synthesized cDNAs were used as templates for RT-PCR. For amplification of conservative domains of retrolelements we used the following primers: LINE_ap3y_F (5'-AAAGCAGGTGGGAGAAACCT-3'), LINE_ap3y_R (5'-GCAACCTTATTGGCTTCACG-3'), LTRB_ap3y_F (5'-TTACACCAAGCAAGGGAAGG-3'), LTRB_ap3y_R (5'-GCAATTACGTGGAAACGACA-3'), LTRA_ap3y_F (5'-AGATTACCCATCGCAACAGG-3'), LTRA_ap3y_R (5'-GACCGAGTATGGCTGGTGTT-3'), IN_ap3y_F (5'-GCGATAGATGCCAACGTTTT-3'), IN_ap3y_R (5'-TTCAAGCCTCTTGCTCCAAT-3'), RT_ap3y_F (5'-TGAGCATCTTCTCGGATTATGT-3'), RT_ap3y_R (5'-TATGCACCGTGTTAGGACCA-3'). PCR conditions were the same as described in DNA amplification. Actin was used as a positive control with primers ActinS-F (5'-CTGCTTACCGAAGCACCATT-3'), ActinS-R (5'-AGGGCGTAACCCTCGTAAAT-3'). The reaction profile for actin included 35 cycles of 30 s at 94°C, 30 sec at 55°C and 30 sec at 72°C preceded by initial denaturation (4 min at 94°C) and followed by final extension step (10 min at 72°C).

### Southern blot hybridization

BAC DNA was restricted by *HindIII *and than transferred by reverse Southern blotting on Hybond N^+ ^(Amersham, Biosciences) membrane filters. Radioactive hybridization was performed as described in the BAC library screening.

### Microdissection

Mitotic slides were prepared according to Lengerova *et al. *[45]. The CellCut Plus system (Olympus) was applied to isolate sex chromosomes and autosomes. Briefly, protoplasts were dropped on a microdissection membrane. Chromosomes of interest were selected and transferred into Eppendorf tube according to manufacturer's protocol (MMI).

### Fluorescence *in situ *hybridization on metaphase chromosomes

Slides were treated as described in Lengerova *et al. *[45] with slight modifications. Slide denaturation was performed in 7:3 (v/v) formamide: 2 X SSC for 2 min at 72°C. Slides were immediately dehydrated through 50%, 70%, and 100% ethanol (-20°C), and air dried. The probe was denatured at 70°C for 10 min, and 100 ng of the denatured probe was added at room temperature and hybridized for 18 h at 37°C. Slides were analyzed using Olympus Provis microscope, and image analysis was performed using ISIS software (Metasystems). DNA was labeled with Fluorolink Cy3-dUTP (Amersham Pharmacia Biotech) (red labeling) in combination with the nick translation mix (Roche).

The probe for the *SlAP3Y *promoter-repeat was synthesized by VBC-Genomics (Vienna) with Cy3 modification on 5'end. The sequence of this probe is: 5'- (ACCCGAN)_3 _-3'.

### Sequencing

BAC DNA was isolated and commercially sequenced from selected BACs using 454 sequencing with Roche GS FLX (GATC Biotech, Konstanz). Basic sequence analysis, sequence assembling and alignment were done with Geneious software. Multiple sequence comparisons were performed with MAFFT [46]http://align.bmr.kyushu-u.ac.jp/mafft/online/server/ and BLAST online applications. A homology search was performed with BLAST [47]. Gene structure identification was done with GENSCAN software [48]http://genes.mit.edu/GENSCAN.html and FGENESH [49]http://linux1.softberry.com/berry.phtml?topic=fgenesh&group=programs&subgroup=gfind.

ORFs were found with ORF Finder [50]http://www.ncbi.nlm.nih.gov/projects/gorf/ and a homology search for conserved domains was performed with NCBI Conserved Domain Search [51]http://www.ncbi.nlm.nih.gov/Structure/cdd/wrpsb.cgi. All primers were designed by Primer3 Input (v.0.4.0) [52]http://frodo.wi.mit.edu/primer3/. Other simple sequence analyses were done by The Sequence Manipulation Suite - version 2 (SMS2), [53]http://www.bioinformatics.org/sms2/.

### Data analysis

To compute the dN and dS between X and Y copies of *SlAP3 *in different dioecious species both GenBank sequences and data from BAC sequencing were used. We then ran codeml with the pairwise option on the X and Y sequences [54]. We also performed a phylogenetic analysis of dN/dS using *SlAP3 *sequences presented in (*S. latifolia *and *S. vulgaris*) and from GenBank (*S. latifolia*, *S. dioica*, *S. diclinis *and *S. conica *from Matsunaga *et al. *[23]). We aligned the sequences using Seaview version 4 [55]. We used the species tree known for the *Silene *species in the alignment [56] instead of the tree built from the alignment because of tree building problems with less than 10 sequences (as in [34], see Additional file 5, figure S5). We then ran codeml (branch model and branch-site options) on the alignment and a tree. Statistical significance was tested using likelihood ratio tests [54]. For the expression analysis, we used EST libraries extracted from *S. latifolia *male and female individuals (Blavet *et al*., in preparation). These libraries are non-normalized, so we estimated the relative gene expression based on the quantity of reads. To estimate the expression of the retrotransposon located in the large intron of *SlAP3Y*, we performed a BLAST search with an E-value cut-off 10^-40 ^versus both male and female libraries.

## Authors' contributions

RC performed *S. vulgaris *BAC library screening, PCRs, fluorescence *in situ *hybridization and bioinformatic analysis of sequences. GABM performed sequence divergence analysis and made substantial contributions to data interpretation. HK performed *S. latifolia *BAC library screening. NB made *in silico *expression analysis of Y linked retroelements. AW made substantial contributions to data interpretation. BV prepared metaphase chromosomes for microdissection and fluorescence *in situ *hybridization. JS and JD constructed *S. vulgaris *BAC library. RH designed the experiments, sorted chromosomes by microdissection and conducted PCR on microdissected chromosomes, helped to interpret the data and drafted the manuscript. All authors read and approved the final manuscript

## Supplementary Material

Additional file 1 **Figure S1 Southern hybridization**. Southern hybridization with BAC DNA restricted using *HindIII*. Individual BAC identifiers are indicated. Different signal intensity is due to differences in the amount of DNA loaded for electrophoresis. Hybridization was carried out with a part of the *SlAP3 *gene covering exons 3-7 as a probe. Click here for file

Additional file 2 **Figure S2 Laser microdissection of the *Silene latifolia *X chromosome and autosomes**. Metaphase protoplasts were dropped on a polyethylene naphthalate membrane and stained with Giemsa. A suitable X chromosome was localized under the inverted microscope (**A**). The membrane was cut around the selected region using a laser microbeam (**B**) and the X chromosome was picked up (**C**) by the adhesive cap of a PCR tube (**D**). Microdissection of autosomes (**E**). Before collecting dissected chromosomes (autosomes), sex chromosomes were removed (burned) by the laser microbeam (**F**). Sex chromosomes are indicated. Click here for file

Additional file 3 **Figure S3 Chromosomal distribution of reverse transcriptase (A) and integrase (B) gene derived probes revealed by FISH experiment**. Metaphase chromosomes of *S. latifolia *male were counterstained with DAPI (blue); the probe was labeled with Cy3-conjugated nucleotides (red). The X and Y chromosomes are indicated, bars indicate 10 μm. Click here for file

Additional file 4 **Figure S4 Specific structure of *SlAP3Y *promoter**. Pink shading shows tandemly arrayed DNA within the promoter. Blue represents a border sequence (inverted repeat) of a hypothetical MITE element. Yellow represents the start of exon1. Click here for file

Additional file 5 **Figure S5 Tree used for the dN/dS analysis**. Tree topology was that of the species phylogeny and branch length has been estimated by PAML. BAC sequences derived data are indicated by asterisk. Click here for file

Additional file 6 **Table S1 Branch-site analysis of SlAP3 sequences**. Click here for file

## References

[B1] Charlesworth D Charlesworth B Marais G Steps in the evolution of heteromorphic sex chromosomes Heredity 2005 95 118 28 10.1038/sj.hdy.6800697 15931241

[B2] Marshall Graves JA Weird animal genomes and the evolution of vertebrate sex and sex chromosomes Annu Rev Genet 2008 42 565 86 10.1146/annurev.genet.42.110807.091714 18983263

[B3] Ming R Moore PH Genomics of sex chromosomes Curr Opin Plant Biol 2007 10 123 30 10.1016/j.pbi.2007.01.013 17300986

[B4] Negrutiu I Vyskot B Barbacar N Georgiev S Monéger F Dioecious plants. A key to the early events of sex chromosome evolution Plant Physiology 2001 127 1418 1424 10.1104/pp.010711 11743084 PMC1540173

[B5] Ainsworth C Boys and girls come out to play: The molecular biology dioecious plants Ann Bot-London 2000 86 211 221 10.1006/anbo.2000.1201

[B6] Blackburn KB Sex chromosomes in plants Nature 1923 112 687 688 10.1038/112687c0

[B7] Winge O On sex chromosomes, sex determination and preponderance of females in some dioecious plants CR Trav Lab Carlsberg 1923 15 1 26

[B8] Vyskot B Hobza R Gender in plants: sex chromosomes are emerging from the fog Trends Genet 2004 20 432 438 10.1016/j.tig.2004.06.006 15313552

[B9] Matsunaga S Hizume M Kawano S Kuroiwa T Cytological analysis in *Melandrium album*: genome size, chromosome size and fluorescence in situ hybridization Cytologia 1994 59 135 149

[B10] Vagera J Paulíková D Doležel J The development of male and female regenerants by in vitro androgenesis in dioecious plant *Melandrium album* Ann Bot 1994 73 455 459 10.1006/anbo.1994.1056

[B11] Veuskens J Ye D Oliveira M Ciupercescu DD Installe P Verhoven HA Negrutiu I Sex determination in the dioecious *Melandrium album *- androgenetic embryogenesis requires the presence of the X-chromosome Genome 1992 35 8 16

[B12] Westergaard M Aberrant Y chromosomes and sex expression in *Melandrium album* Hereditas 1946 32 419 443 10.1111/j.1601-5223.1946.tb02784.x 20998142

[B13] Westergaard M The mechanism of sex determination in dioecious flowering plants Adv Genet 1958 9 217 281 full_text 13520443 10.1016/s0065-2660(08)60163-7

[B14] Farbos I Veuskens J Vyskot B Oliveira M Hinnisdaels S Aghmir A Mouras A Negrutiu I Dimorphism in white campion: Deletion on the Y chromosome results in afloral asexual phenotype Genetics 1999 151 1187 1196 10049934 10.1093/genetics/151.3.1187 PMC1460540

[B15] Lebel-Hardenack S Hauser E Law TF Schmid J Grant SR Mapping of sex determination loci on the white campion (*Silene latifolia*) Y chromosome using amplified fragment length polymorphism Genetics 2002 160 717 25 11861573 10.1093/genetics/160.2.717 PMC1461999

[B16] Bergero R Charlesworth D Filatov DA Moore RC Defining regions and rearrangements of the *Silene latifolia *Y chromosome Genetics 2008 178 2045 53 10.1534/genetics.107.084566 18245827 PMC2323795

[B17] Babushok DV Ostertag EM Kazazian HH Jr Current topics in genome evolution: molecular mechanisms of new gene formation Cell Mol Life Sci 2007 64 542 54 10.1007/s00018-006-6453-4 17192808 PMC11138463

[B18] Kaessmann H Vinckenbosch N Long M RNA-based gene duplication: mechanistic and evolutionary insights Nat Rev Genet 2009 10 19 31 10.1038/nrg2487 19030023 PMC3690669

[B19] Lahn BT Pearson NM Jegalian K The human Y chromosome, in the light of evolution Nat Rev Genet 2001 2 207 216 10.1038/35056058 11256072

[B20] Emerson JJ Kaessmann H Betrán E Long M Extensive gene traffic on the mammalian X chromosome Science 2004 303 537 40 10.1126/science.1090042 14739461

[B21] Kejnovsky E Hobza R Kubat Z Cermak T Vyskot B The role of repetitive DNA in structure and evolution of sex chromosomes in plants Heredity 2009 102 533 541 10.1038/hdy.2009.17 19277056

[B22] Bachtrog D Expression profile of a degenerating neo-Y chromosome in *Drosophila* Curr Biology 2006 16 1694 1699 10.1016/j.cub.2006.07.053 16950105

[B23] Matsunaga S Isono E Kejnovsky E Vyskot B Kawano S Charlesworth D Duplicative transfer of MADS box gene to a plant Y chromosome Mol Biol Evol 2003 20 1062 1069 10.1093/molbev/msg114 12716981

[B24] Zluvova J Janousek B Negrutiu I Vyskot B Comparison of the X and Y chromosome organization in *Silene latifolia* Genetics 2005 170 1431 4 10.1534/genetics.105.040444 15879508 PMC1451171

[B25] Nicolas M Marais G Hykelova V Janousek B Laporte V Vyskot B Mouchiroud D Negrutiu I Charlesworth D Monéger F A gradual process of recombination restriction in the evolutionary history of the sex chromosomes in dioecious plants PloS Biol 2005 3 47 56 10.1371/journal.pbio.0030004 PMC536007 15630476

[B26] Kejnovsky E Kubat Z Macas J Hobza R Mracek J Vyskot B A novel family of gypsy-like retrotransposons harboring an amplified tandem repeat Molecular Genetics and Genomics 2006 276 254 263 10.1007/s00438-006-0140-x 16826419

[B27] Matsunaga S Kawano S Takano H Uchida H Sakai A Kuroiwa T Isolation and developmental expression of male reproductive organ-specific genes in a dioecious campion, Melandrium album (*Silene latifolia*) Plant J 1996 10 679 89 10.1046/j.1365-313X.1996.10040679.x 8893544

[B28] Bergero R Forrest A Kamau E Charlesworth D Evolutionary strata on the X chromosomes of the dioecious plant *Silene latifolia*: evidence from new sex-linked genes Genetics 2007 175 1945 54 10.1534/genetics.106.070110 17287532 PMC1855140

[B29] Kejnovský E Vrána J Matsunaga S Soucek P Siroký J Dolezel J Vyskot B Localization of male-specifically expressed MROS genes of *Silene latifolia *by PCR on flow-sorted sex chromosomes and autosomes Genetics 2001 158 1269 77 11454773 10.1093/genetics/158.3.1269 PMC1461734

[B30] Hobza R Hrusakova P Safar J Bartos J Janousek B Zluvova J Michu E Dolezel J Vyskot B MK17, a specific marker closely linked to the gynoecium suppression region on the Y chromosome in *Silene latifolia* Theoretical and Applied Genetics 2006 113 280 287 10.1007/s00122-006-0293-3 16791694

[B31] Hobza R Vyskot B Laser microdissection- based analysis of plant sex chromosomes Methods Cell Biol 2007 82 433 53 full_text 17586267 10.1016/S0091-679X(06)82015-7

[B32] Wilson MA Makova KD Evolution and survival on eutherian sex chromosomes PloS Genet 2009 5 e1000568 10.1371/journal.pgen.1000568 19609352 PMC2704370

[B33] Moore RC Kozyreva O Lebel-Hardenack S Siroky J Hobza R Vyskot B Grant SR Genetic and functional analysis of DD44, a sex-linked gene from the dioecious plant *Silene latifolia*, provides clues to early events in sex chromosome evolution Genetics 2003 163 321 334 12586719 10.1093/genetics/163.1.321 PMC1462427

[B34] Marais G Nicolas M Bergero R Chambrier P Kejnovsky E Monéger F Hobza R Widmer A Charlesworth D Evidence for degeneration of the Y chromosome in the dioecious plant *Silene latifolia* Current Biology 2008 18 545 549 10.1016/j.cub.2008.03.023 18394889

[B35] Kubat Z Hobza R Vyskot B Kejnovsky E Microsatellite accumulation on the Y chromosome in *Silene latifolia* Genome 2008 51 350 356 10.1139/G08-024 18438438

[B36] Hobza R Lengerova M Svoboda J Kubekova H Kejnovsky E Vyskot B An accumulation of tandem DNA repeats on the Y chromosome in *Silene latifolia *during early stages of sex chromosome evolution Chromosoma 2006 115 376 382 10.1007/s00412-006-0065-5 16612641

[B37] Kejnovsky E Kubat Z Hobza R Lengerova M Sato I Tabata S Fukui K Matsunaga S Vyskot B Accumulation of chloroplast DNA sequences on the Y chromosome of Silene latifolia Genetica 2006 128 167 175 10.1007/s10709-005-5701-0 17028949

[B38] Cermak T Kubat Z Hobza R Koblizkova A Widmer A Macas J Vyskot B Kejnovsky E Survey of repetitive sequences in *Silene latifolia *with respect to their distribution on sex chromosomes Chromosome Research 2008 16 961 976 10.1007/s10577-008-1254-2 18853265

[B39] Filatov DA Howell EC Groutides C Armstrong SJ Recent spread of a retrotransposon in the *Silene latifolia *genome, apart from the Y chromosome Genetics 2009 181 811 7 10.1534/genetics.108.099267 19064703 PMC2644968

[B40] Pasyukova E Nuzhdin S Li W Flavell AJ Germ line transposition of the copia retrotransposon in *Drosophila melanogaster *is restricted to males by tissue-specific control of copia RNA levels Mol Gen Genet 1997 255 115 24 10.1007/s004380050479 9230904

[B41] Matzke MA Birchler JA RNAi-mediated pathways in nucleus Nat Rev Genet 2005 6 24 35 10.1038/nrg1500 15630419

[B42] Bouzidi MF Franchel J Tao Q Stormo K Mraz A Nicolas P Mouzeyar S A sunflower BAC library suitable for PCR screening and physical mapping of targeted genomic regions Theor Appl Genet 2006 113 81 9 10.1007/s00122-006-0274-6 16783592

[B43] Široký J Lysák MA Doležel J Kejnovský E Vyskot B Heterogeneity of rDNA distribution of genome size in *Silene spp* Chromosome Research 2001 9 387 393 10.1023/A:1016783501674 11448040

[B44] Sambrook J Russell DW Molecular cloning: a laboratory manual 2001 3 Cold Spring Harbor, New York; Cold Spring Harbor Laboratory Press

[B45] Lengerova M Kejnovsky E Hobza R Macas J Grant SR Vyskot B Multicolor FISH mapping of the dioecious model plant, *Silene latifolia* Theor Appl Genet 2004 108 1193 1199 10.1007/s00122-003-1568-6 14727034

[B46] Katoh Asimenos Toh Multiple Alignment of DNA Sequences with MAFFT Bioinformatics for DNA Sequence Analysis edited by D. Posada (outlines DNA alignment methods and several tips including group-to-group alignment and rough clustering of a large number of sequences). Methods in Molecular Biology 2009 537 39 64 10.1007/978-1-59745-251-9_3 19378139

[B47] Stephen F David J Basic local alignment search tool J Mol Biol 1990 215 403 410 2231712 10.1016/S0022-2836(05)80360-2

[B48] Burge C Karlin S Prediction of complete gene structures in human genomic DNA J Mol Biol 1997 268 78 94 10.1006/jmbi.1997.0951 9149143

[B49] Salamov AA Solovyev VV Ab initio gene finding in Drosophila genomic DNA Genome Res 2000 10 516 522 10.1101/gr.10.4.516 10779491 PMC310882

[B50] Tatusov T Tatusov R ORF Finder (Open Reading Frame Finder) National Center for Biotechnology Information. National Institute of Health 2007 http://www.ncbi.nlm.nih.gov/projects/gorf/

[B51] Marchler-Bauer A Bryant SH "CD-Search: protein domain annotations on the fly" Nucleic Acids Res 2004 32 327 331 10.1093/nar/gkh454 PMC441592 15215404

[B52] Rozen S Skaletsky HJ Krawetz S, Misener S Primer3 on the WWW for general users and for biologist programmers Bioinformatics Methods and Protocols Methods in Molecular Biology 2000 Humana Press, Totowa, NJ 365 386 10.1385/1-59259-192-2:365 10547847

[B53] Stothard P The Sequence Manipulation Suite: JavaScript programs for analyzing and formatting protein and DNA sequences Biotechniques 2000 28 1102 1104 10868275 10.2144/00286ir01

[B54] Yang Z PAML 4: phylogenetic analysis by maximum likelihood Mol Biol Evol 2007 24 1586 91 10.1093/molbev/msm088 17483113

[B55] Gouy M Guindon S Gascuel O SeaView version 4 A multiplatform graphical user interface for sequence alignment and phylogenetic tree building Mol Biol Evol 2010 27 221 4 10.1093/molbev/msp259 19854763

[B56] Desfeux C Maurice S Henry JP Lejeune B Gouyon PH Evolution of reproductive systems in the genus Silene Proc Biol Sci 1996 263 409 14 Ainsworth C: **Boys and girls come out to play: The molecular biology dioecious plants**. Ann Bot-London 2000, 86: 211-221 10.1098/rspb.1996.0062 18386410

