# Supplementary figures and images for: Structure and evolution of Apetala3, a sex-linked gene in Silene latifolia
Source: BMC Plant Biol. 2010 Aug 18;10:180. doi: 10.1186/1471-2229-10-180 (PMC3095310; doi:10.1186/1471-2229-10-180)

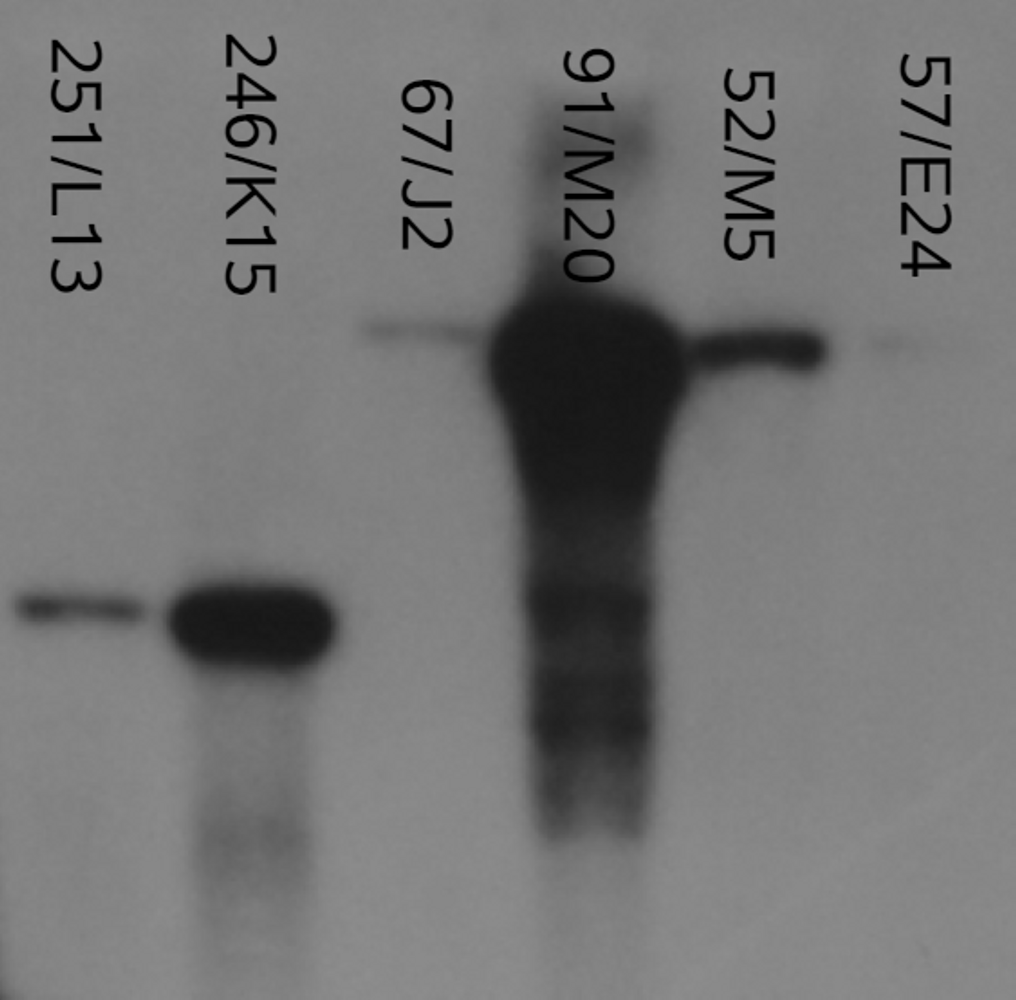

Supplement: Additional file 1 — Figure S1 Southern hybridization. Southern hybridization with BAC DNA restricted using HindIII. Individual BAC identifiers are indicated. Different signal intensity is due to differences in the amount of DNA loaded for electrophoresis. Hybridization was carried out with a part of the SlAP3 gene covering exons 3-7 as a probe. [file 1471-2229-10-180-S1.PNG]

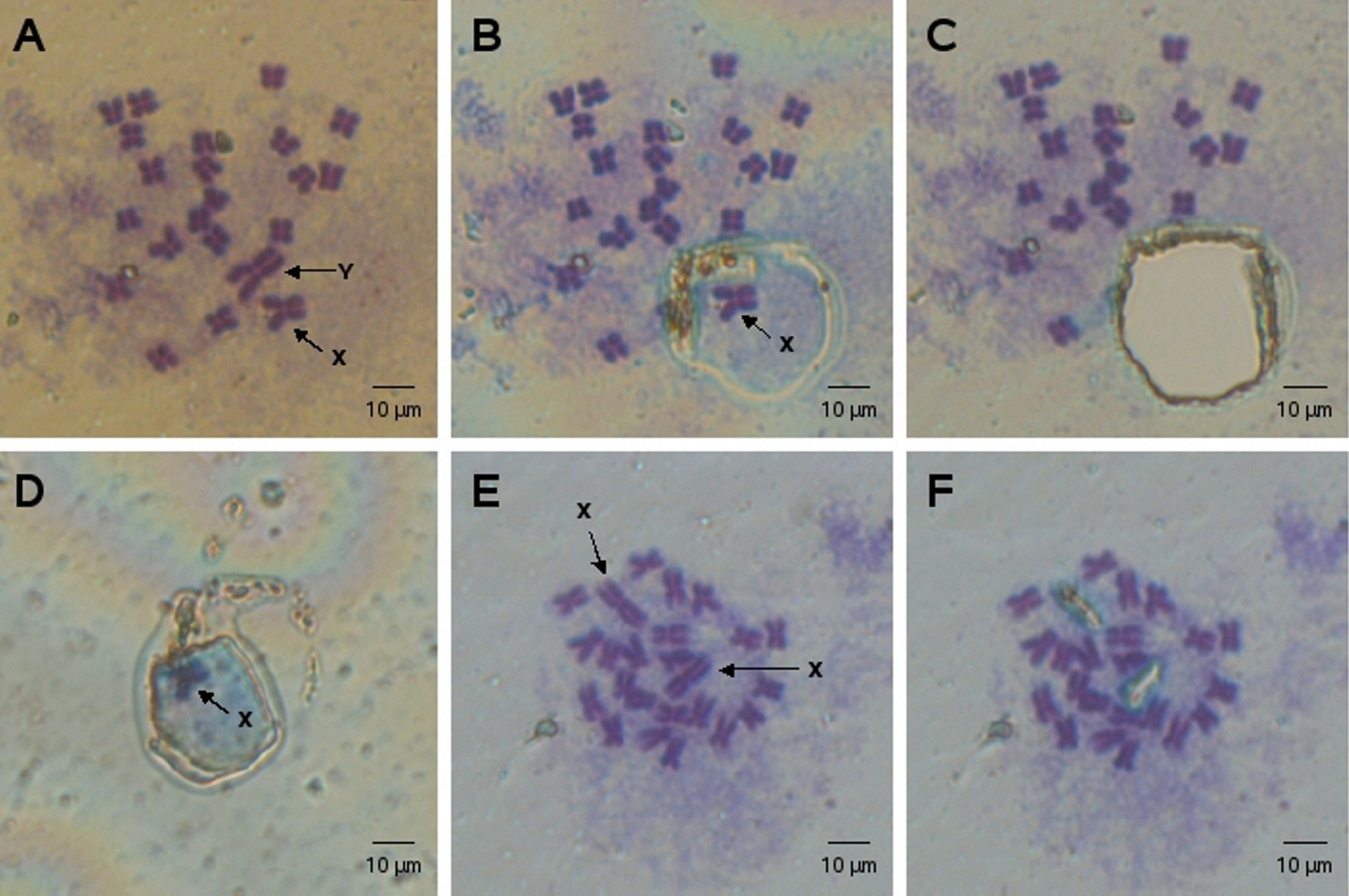

Supplement: Additional file 2 — Figure S2 Laser microdissection of the Silene latifolia X chromosome and autosomes. Metaphase protoplasts were dropped on a polyethylene naphthalate membrane and stained with Giemsa. A suitable X chromosome was localized under the inverted microscope (A). The membrane was cut around the selected region using a laser microbeam (B) and the X chromosome was picked up (C) by the adhesive cap of a PCR tube (D). Microdissection of autosomes (E). Before collecting dissected chromosomes (autosomes), sex chromosomes were removed (burned) by the laser microbeam (F). Sex chromosomes are indicated. [file 1471-2229-10-180-S2.PNG]

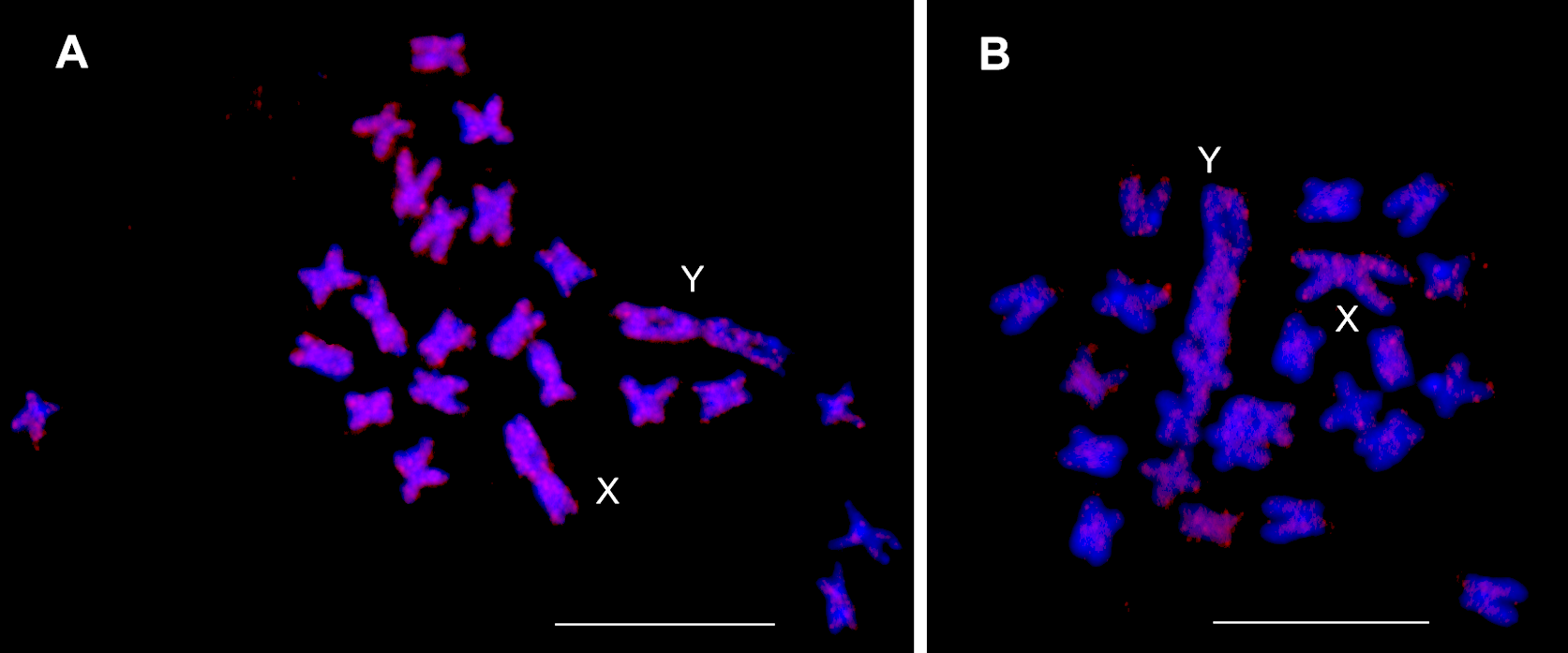

Supplement: Additional file 3 — Figure S3 Chromosomal distribution of reverse transcriptase (A) and integrase (B) gene derived probes revealed by FISH experiment. Metaphase chromosomes of S. latifolia male were counterstained with DAPI (blue); the probe was labeled with Cy3-conjugated nucleotides (red). The X and Y chromosomes are indicated, bars indicate 10 μm. [file 1471-2229-10-180-S3.PNG]

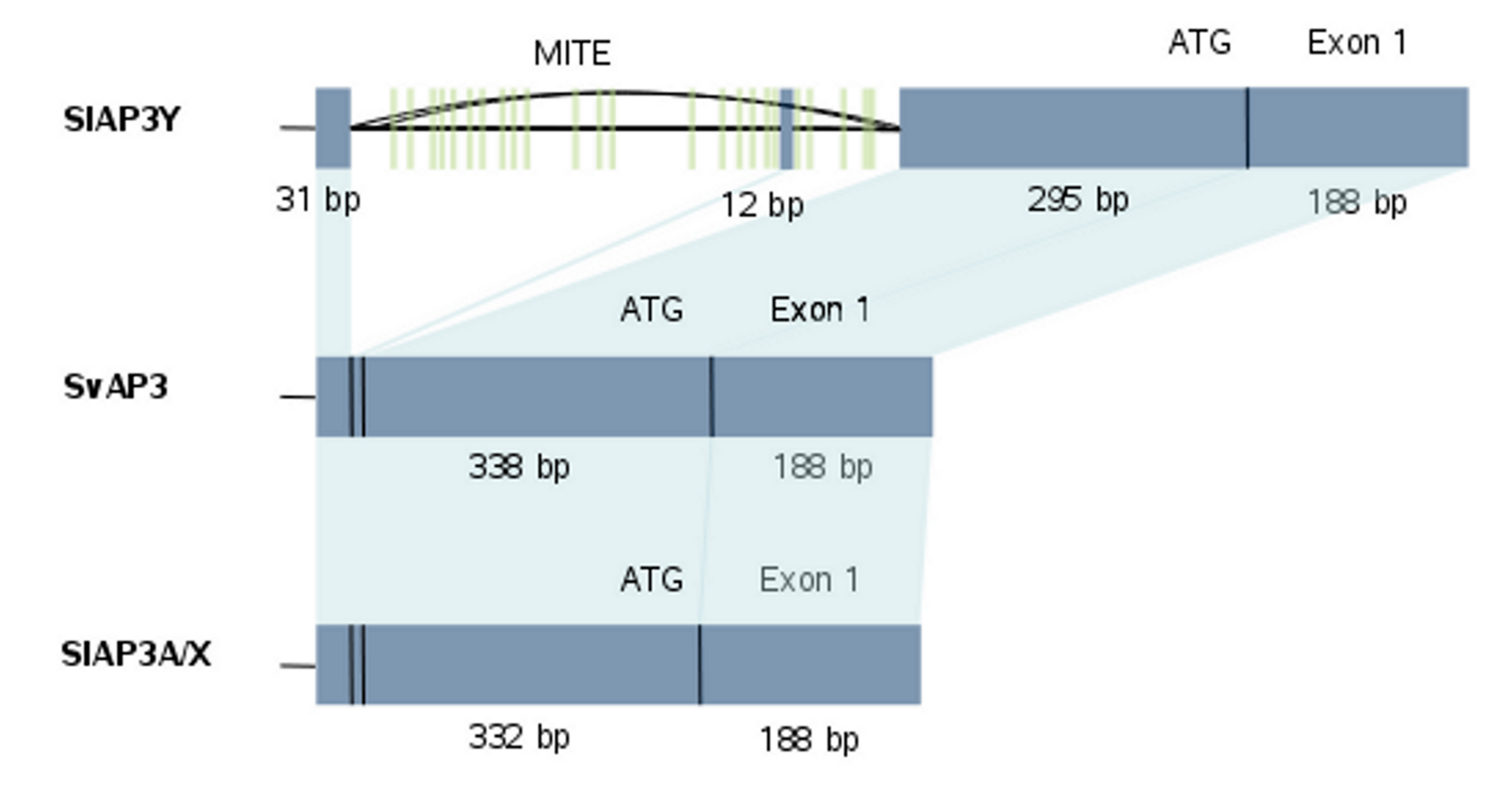

Supplement: Additional file 4 — Figure S4 Specific structure of SlAP3Y promoter. Pink shading shows tandemly arrayed DNA within the promoter. Blue represents a border sequence (inverted repeat) of a hypothetical MITE element. Yellow represents the start of exon1. [file 1471-2229-10-180-S4.PNG]

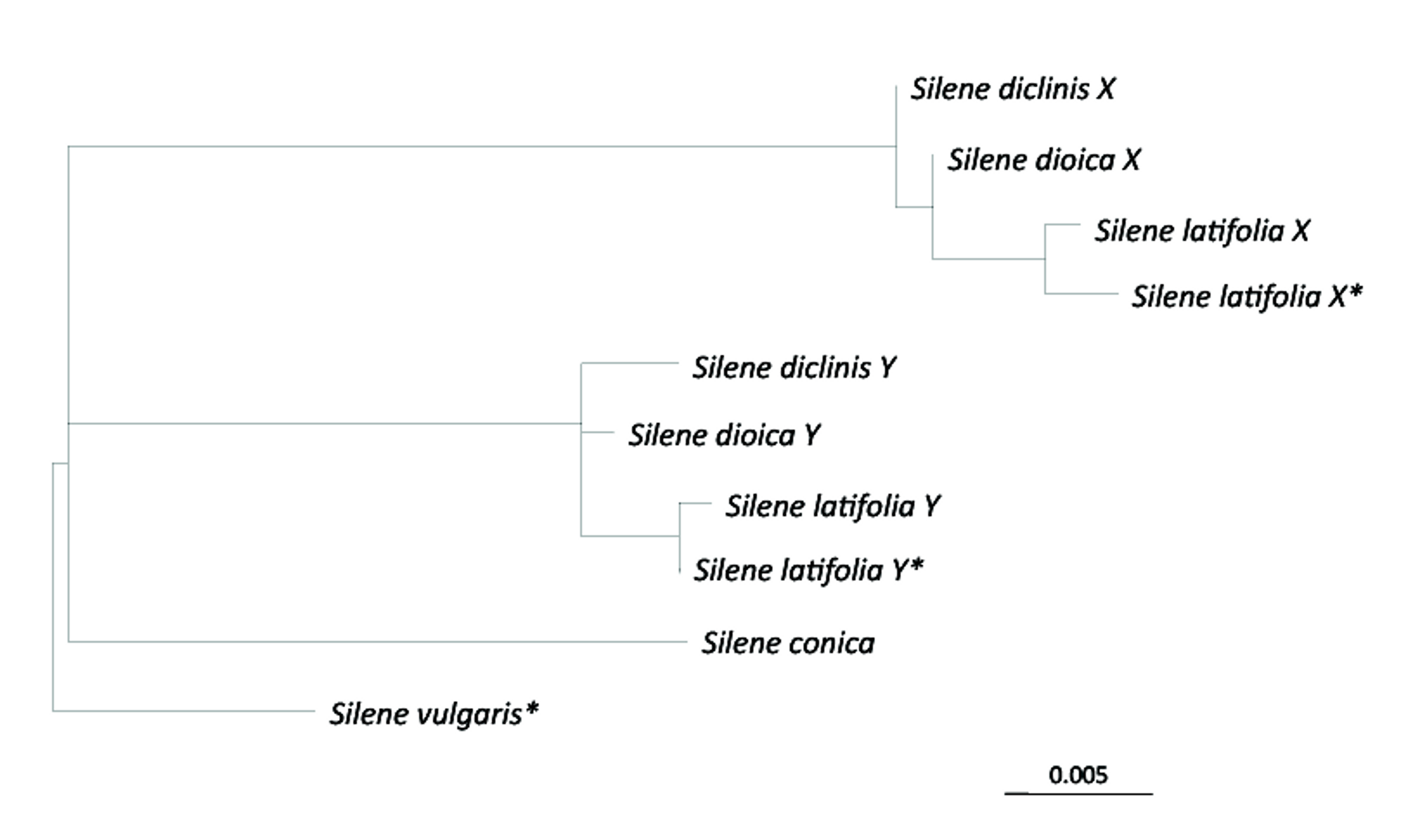

Supplement: Additional file 5 — Figure S5 Tree used for the dN/dS analysis. Tree topology was that of the species phylogeny and branch length has been estimated by PAML. BAC sequences derived data are indicated by asterisk. [file 1471-2229-10-180-S5.PNG]
