# Supplementary material for: Structure and evolution of Apetala3, a sex-linked gene in Silene latifolia
Source: BMC Plant Biol. 2010 Aug 18;10:180. doi: 10.1186/1471-2229-10-180 (PMC3095310; doi:10.1186/1471-2229-10-180)
Supplement: Additional file 6 — Table S1 Branch-site analysis of SlAP3 sequences. [file 1471-2229-10-180-S6.DOCX]

| Fast-evolving codons | In Y sequences | In X sequences | In both X and Y sequences |
| --- | --- | --- | --- |
| Fraction | 9.2% | 11.5% | 19.8% |
| dN/dS | 1 | 2.54 | 1.49 |
| LRT (H0: dN/dS = 1) | - | Not significant | Not significant |
